# Global Array-Based Transcriptomics from Minimal Input RNA Utilising an Optimal RNA Isolation Process Combined with SPIA cDNA Probes

**DOI:** 10.1371/journal.pone.0017625

**Published:** 2011-03-22

**Authors:** Laura Kennedy, Mahesh Pauriah, Valerie Godfrey, Jacqueline Howie, Helen Dennis, Daniel Crowther, Allan Struthers, Catharine Goddard, Giora Feuerstein, Chim Lang, Gino Miele

**Affiliations:** 1 Translational Medicine Research Collaboration, TMRC Laboratory, Ninewells Hospital, Dundee, United Kingdom; 2 Centre for Cardiovascular and Lung Biology, Division of Medical Sciences, Ninewells Hospital and Medical School, University of Dundee, Dundee, United Kingdom; 3 Pfizer Global Research & Development, Collegeville, Pennsylvania, United States of America; 4 Pfizer Global Research & Development, Dundee, United Kingdom; University of Cambridge, United Kingdom

## Abstract

Technical advances in the collection of clinical material, such as laser capture microdissection and cell sorting, provide the advantage of yielding more refined and homogenous populations of cells. However, these attractive advantages are counter balanced by the significant difficultly in obtaining adequate nucleic acid yields to allow transcriptomic analyses. Established technologies are available to carry out global transcriptomics using nanograms of input RNA, however, many clinical samples of low cell content would be expected to yield RNA within the picogram range. To fully exploit these clinical samples the challenge of isolating adequate RNA yield directly and generating sufficient microarray probes for global transcriptional profiling from this low level RNA input has been addressed in the current report. We have established an optimised RNA isolation workflow specifically designed to yield maximal RNA from minimal cell numbers. This procedure obtained RNA yield sufficient for carrying out global transcriptional profiling from vascular endothelial cell biopsies, clinical material not previously amenable to global transcriptomic approaches. In addition, by assessing the performance of two linear isothermal probe generation methods at decreasing input levels of good quality RNA we demonstrated robust detection of a class of low abundance transcripts (GPCRs) at input levels within the picogram range, a lower level of RNA input (50 pg) than previously reported for global transcriptional profiling and report the ability to interrogate the transcriptome from only 10 pg of input RNA. By exploiting an optimal RNA isolation workflow specifically for samples of low cell content, and linear isothermal RNA amplification methods for low level RNA input we were able to perform global transcriptomics on valuable and potentially informative clinically derived vascular endothelial biopsies here for the first time. These workflows provide the ability to robustly exploit ever more common clinical samples yielding extremely low cell numbers and RNA yields for global transcriptomics.

## Introduction

Microarray technologies permitting global gene expression profiling have represented a major advance in genomic science. Gene expression profiles can generate parallel quantitative and repeated measurements of tens of thousands of transcripts simultaneously in one sample, allowing rational comparison of the transcriptome across a vast array of experimental conditions. Several advances in RNA amplification techniques and microarray technologies have led to the wide use of global gene expression profiling in biomedical research, adopted as standard in both academia and industry [1], [2], [3]. These capabilities can frequently provide powerful insights into biological processes underlying disease and through translational research can underpin generation of target gene modules for potential use in molecular disease diagnosis and prognosis, and in patient stratification schema and prediction of therapeutic outcome in personalised medicine strategies [4], [5].

Historically, microarray probe generation has been achieved by *in vitro* transcription (IVT) with T7 RNA polymerase of double-stranded cDNA, firstly generated by reverse-transcription of mRNA utilising a poly(T) oligonucleotide complementary to the 3′ poly(A) tail of mRNA (or internal poly(A) tracts) [6], requiring total RNA input within the microgram range [7], [8]. Advances in sample collection techniques such as fine needle aspiration, laser capture micro-dissection (LCM) and cell sorting now provide the opportunity to interrogate the transcriptome of more homogenous, refined cell populations. However, these types of material are far more limited in quantity leading to greatly reduced RNA yields. Early attempts at using fine needle aspirates for microarray based global gene expression profiling have been largely unsuccessful due to only a small percentage (∼15%) of samples providing sufficient RNA quantities required for the probe generation methods of the time [9]. These studies were subsequently followed by a more targeted approach allowing assessment of a small number of pre-defined target mRNAs [9], [10]. The few studies which have reported successful profiling from fine needle aspiration biopsies have done so using biopsies from tumour samples which provide at least 1 µg of total RNA to use as input for probe generation [11].

More targeted approaches to gene expression profiling, such as real-time quantitative PCR (qPCR), have the advantage of conceptually requiring less input material and indeed are vital in validating gene expression profiles identified and inferred from array-based global transcriptome datasets and as the molecular platform in standard nucleic acid molecular diagnostics. qPCR used along side advances in microfluidics technology has demonstrated the possibility of gene expression analysis at the single cell level [12], [13]. However, qPCR technologies, regardless of sensitivity, are limited to providing expression levels of predetermined genes and as such, inherently restrict the potential for novel discovery. Information from greater numbers of transcripts can be generated using the technique of poly-A tailed mRNA sequencing, however, commercially available kits for this technique still require up to micrograms of input material. The growing potential of sequencing to carry out transcriptomics has been recently demonstrated by reports of this technology being used at the single cell level [14].

In response to growing requirements to carry out global gene expression profiling on limited sample material further advances in RNA amplification have arisen, providing numerous technologies claiming the ability to process RNA amounts within the nanogram range [15], [16], [17], [18]. Using these methods several groups have reported global transcriptomics data from LCM and flow cytometry collected cells by *in vitro* transcription-based methods using 100 and 200 ng of input RNA, respectively, followed by hybridisation to Affymetrix GeneChips [19], [20].

Continuing technical advances in sample collection or dissection have consequently resulted in informative interrogation of important clinical material comprised of hundreds or thousands of cells rather than from millions as was previously possible [21], [22]. However, some attempts at microarray experiments using these clinical samples of reduced cell content have either pooled individual samples to gain sufficient RNA [21] or focussed on optimising the crucial RNA isolation step to obtain the nanograms of RNA required for microarray analysis [22]. Microarray analysis of 1000 endothelial cells reported the robust detection of only hundreds of transcripts rather than the several thousands expected from a successful and informative microarray experiment [23], indicating successful detection of only the most abundant mRNAs in the limited material. Given the obvious difficulties in obtaining nanograms of RNA following direct extraction from minimal cell numbers, or capturing the valuable inherent biological variation following pooling of samples, there is a clear requirement for RNA amplification methods allowing microarray probe generation from within the picogram range of RNA to allow such investigations.

Whilst there are reports comparing RNA amplification methods which have successfully carried out microarray gene expression profiling from picograms of RNA [17], [24], [25], many have utilised titrated dilutions of stock total RNA to within the picogram range as the basis of support for the capabilities of the probe generation technologies documented. However, importantly, none of these address the issue of retrieving this level of RNA directly from minimal cell samples such that it is applicable to microarray workflows [17], [24]. Microarray data has been obtained using RNA isolated directly from single embryonic stem cell (ESC) colonies, however, the authors do estimate that a single ESC colony will yield nanograms of RNA [26]. Although possible to generate data from single ESC colonies, in practice it was suggested that pooling of several colonies was required to generate reliable results [26].

Successful microarray analysis on RNA isolated from minimal cell samples has been previously reported. ESC colonies [26], 100 flow-sorted lymphocytes [27] and even individual human oocytes and embryos, thought to yield 55 pg and 20 pg RNA, respectively [28] have been subjected to microarray interrogation. These reports are all based on amplification methods of two successive rounds of amplification using 3′ priming, and in some cases three successive rounds [6]. These methods thereby potentially introduce significant 3′ and abundance bias, and can also resulted in relatively low overall representation of the transcriptome [27]. The extent of transcriptome bias introduced by the extensive 3′ primed amplification of individual oocyte or embryo templates is also unknown, with reported reproducibility data from unamplified versus amplified samples restricted to a subset of genes (∼5000) produced using 500 pg of RNA [28].

The ability to extract adequate material for global molecular analyses from smaller clinical samples paves the way for less invasive sample collection techniques within the clinic, for example a ‘finger pick’ for blood versus a full phlebotomy procedure. Small clinical specimens also have specific value in translational medicine where collection is more permissive to repeated sampling, facilitating longitudinal biomarker discovery and validation assessments.

One clinical sample type for which the ability to carry out transcriptional profiling (global or targeted) has previously been significantly hampered due to lack of cell numbers and RNA yield but could lead to significant advancements in understanding disease mechanisms, patient stratification and disease progression are vascular endothelial cell scraping biopsies [29]. Vascular endothelial dysfunction plays a major role in the pathogenesis of metabolic and cardiovascular disease [30], [31], [32] and further characterisation of the vascular endothelium prior to and during disease progression could prove useful in understanding risk factors, disease mechanisms and identification of potential therapeutic intervention strategies. We and others have therefore been keen to identify molecular markers indicative and perhaps predictive of endothelial dysfunction. To facilitate access, a minimally invasive technique to safely collect vascular endothelial cell biopsies from either a superficial forearm vein or the radial artery in human subjects has been previously established [29]. Protein expression measurements by quantitative immunofluorescence and immunoblotting have been carried out on these minimal endothelial cell samples [29], [33], along with restricted, targeted gene expression analyses [34]. However, due to the low number of endothelial cells collected (mean of ∼97 cells in the current study) and the subsequent low RNA yield these types of material have never been practically accessible for global gene expression profiling. Considering the potential impact of obtaining high quality and faithful global gene expression profiles using picograms of input RNA from these vascular endothelial cell biopsies, we first sought to establish a consistent, robust and practical workflow to allow the application of these to global profiling.

In order to guarantee maximum RNA yield from these minimal cell samples, an optimised RNA isolation workflow was established specifically designed to minimise RNA loss throughout the procedure, whilst still being amenable to downstream array probe generation. For successful microarray analysis unbiased and efficient amplification of this RNA is also required. The WT-Ovation RNA amplification systems (NuGEN Technologies™, USA) used for cDNA probe generation are commonly mentioned in reports of microarray gene expression profiling using picograms of RNA [17], [24], [27]. The Ovation systems utilise a single primer, isothermal linear amplification (SPIA) method [17], [35] to generate single-strand cDNA microarray probes suitable for use with Affymetrix GeneChips™ among other platforms. Reproducibility studies and comparison with other RNA amplification methods have illustrated a high degree of consistence and greater hybridisation specificity when exploiting sscDNA:DNA hybridisation compared to cRNA:DNA on microarrays [36] and reported RNA input amounts of 250 pg using versions of this technology [24]. More recent members of the WT-Ovation RNA amplification system family may now provide further opportunity to exploit minimal cell samples. The WT-Ovation FFPE RNA amplification system V2, although principally designed to amplify sscDNA probes from the highly degraded/fragmented and modified RNA obtained from FFPE (formalin fixed paraffin embedded) material, has increased SPIA amplification capacity which can conceptually be harnessed for use with samples of good quality RNA but in low picogram quantities. Further advances in WT-Ovation amplification technology have come in the form of the NuGEN Technologies™ WT-Ovation™ One-Direct RNA amplification system. This system is reported to have the increased RNA amplification potential to synthesise adequate cDNA probe from as little as 10 pg RNA. Both of these technologies were assessed here for performance using both varying amounts of template RNA titrated from stocks and RNA yielded from clinically-derived vascular endothelial cell biopsies.

In the present study we identify the optimal workflow which allows (a) efficient RNA isolation from minimal cell numbers, (b) efficient microarray probe generation from picograms of input RNA which allows the continued detection of a rare class of transcripts (widely reported to be challenging to detect by array technology) over a range of abundances and (c) the generation of global gene expression profiles from clinically relevant vascular endothelial biopsies previously restricted to focussed interrogation.

## Results

### Optimisation of RNA isolation from minimal cell numbers

**Figure 1:**
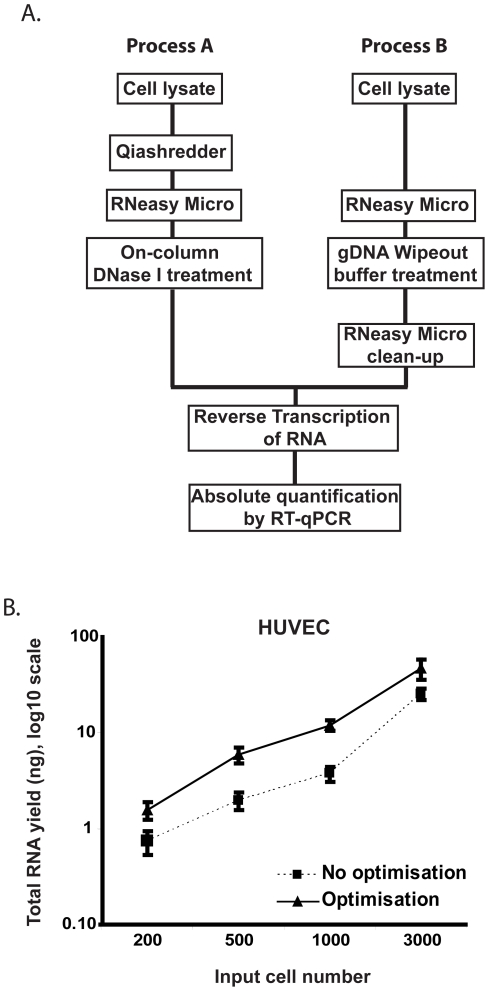
Comparison of experimental workflows for optimised total RNA isolation from minimal cell numbers. (**A**) Titrated HUVEC samples were processed for RNA isolation through Process A or B to establish the optimal workflow for RNA yield from minimal cell numbers. (**B**) HUVECs were titrated over a range of 3000 - 200 cells per tube and split in to 6 aliquots for cell lysis and RNA isolation. Triplicate samples were processed using either process A or B. All samples were then quantified by reverse transcription of the entire yielded RNA and real-time quantitative PCR using SybrGreen probe and β-actin primers against a standard curve of known HUVEC RNA input. Error bars  =  standard deviation.

Human umbilical venous endothelial cells represent an *in vitro* “analogue” of the human vascular endothelial cell biopsies utilised in this study [29]. To quantify the effect of various adaptations to the RNA isolation workflow on RNA yield from minimal cell numbers, reverse transcription followed by real-time quantitative PCR was carried out against a calibration set processed and prepared in parallel utilising titrated samples of HUVEC cells (3000-200 cells) (Figure 1A).

Optimisation of the RNA isolation workflow included the assessment of several carrier molecules (bacterial rRNA, yeast tRNA, poly(I)(C), linear acrylamide) with combinations of Isopropanol/Ethanol precipitation with ammonium acetate to aid in RNA yield retention compared to commercial column-based isolation techniques (Qiagen RNeasy Micro). Only one carrier (poly(A) RNA) was shown to be advantageous in final yields of RNA under the conditions assessed, but is unfortunately incompatible with downstream array probe synthesis techniques (data not shown). The RNeasy column-based technologies can incorporate optional QIAshredder™ homogenisation of the cell lysate and an on-column DNase I treatment to eliminate genomic DNA from subsequent RNA samples. In a series of preliminary investigations, QIAshredder cell lysate homogenisation at low levels of input cells, <3000 cells was shown to be detrimental to the resultant RNA yield compared to vigorous vortexing (data not shown). On-column DNase I treatment also resulted in an overall 20% loss in RNA yield (data not shown). As an alternative to enzymatic elimination of genomic DNA, gDNA Wipeout reagent (Qiagen) has been demonstrated to result in genomic DNA free-RNA, even from input template amounts of 1 µg RNA with moderate gDNA contamination. Whilst we were able to verify no negative effect on RNA yield of introduction of this step using this proprietary reagent, it was also apparent that the resultant RNA samples containing gDNA Wipeout reagent were incompatible with downstream probe synthesis technologies, thus necessitating removal by further purification schema.

Analysis of the resultant cDNA from both process A and following optimisation, process B by comparison to a reference HUVEC RNA calibration set clearly demonstrated that optimisation of the RNA isolation workflow resulted in significantly increased yields of isolated RNA (approximately 2–3 fold higher), particularly important at the 200 cell range (Figure 1B, Table 1). We therefore proceeded with this methodology, yielding on average 8 pg total RNA per HUVEK cell, for downstream microarray probe generation workflows, to achieve global transcriptomics from input RNA in the low picogram range.

**Table 1:** RNA yield from titrated HUVEC following RNA isolation using process A or process B.

Cell input	Process A RNA yield (ng)	Process B RNA Yield (ng)
3000	25.2±(3.5)	46.1±(11.3)
1000	3.8±(0.7)	11.9±(1.5)
500	2.0±(0.4)	5.9+(1.1)
200	0.8±(0.2)	1.6+(0.3)

Cells were titrated over a range of 3000-200 cells per tube and each split equally into 6 aliquots for subsequent cell lysis and RNA isolation by either of two process (Process A or B). All resultant RNAs were quantified by reverse transcription of the entire yielded RNA and quantitative real-time PCR against a standard curve of known HUVEC total RNA input. The table shows mean total RNA yields (numbers in parenthesis  =  standard deviation from three technical replicates).

### Total RNA isolation and yield from vascular endothelial cell biopsies

Vascular endothelial cell biopsy clinical samples were collected following written informed consent from 114 patients (cardiovascular disease and healthy controls) in a study that had been approved by the local ethics committee. Biological interpretation of these data and accompanying clinical observations will be reported elsewhere. All samples were processed through the optimal RNA isolation workflow outlined here (process B). Assessment of yielded RNA was performed by converting 10% of the yielded RNA to cDNA in parallel with a HUVEK cell RNA standard curve for comparative assessment using β-Actin qCPR. The average yield across all 114 samples was 1032.6±848.6 pg (mean ± standard deviation), with a median of 778 pg and an absolute range of 73 pg to 5027 pg of total RNA. Of the 114 clinical biopsies, 36.8% yielded over 1 ng and 28.1% of samples yielded less than 500 pg of RNA. With the assumption that RNA content per cell is similar in purified endothelial cells as in HUVEK cells, our approximations of numbers of cells yielded in the collection procedure equates to a range of 9 to 638 cells, with a median of 97 cells. These data illustrate the requirement for a microarray workflow capable of operation at below 500 pg to maximise cohort utility and successfully interrogate vascular endothelial biopsies by microarray technology.

### Efficacy of cDNA probe generation from minimal total RNA input

The efficacies of WT-Ovation FFPE RNA amplification system V2 and WT-Ovation One-Direct RNA amplification system were assessed using RNA template input within the picogram range.

**Figure 2:**
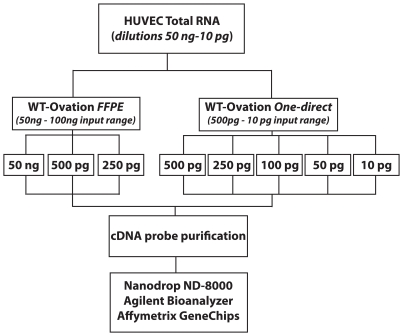
Experimental workflow to assess efficiency of NuGen probe generation technologies using low amounts of input RNA. HUVEC total RNA was titrated to cover a range of input RNA from 50 ng–10 pg. 50 ng (n = 1), 500 pg (n = 2) and 250 pg (n = 2) of total RNA was used as input for the WT-Ovation FFPE system V2 while 500 pg (n = 2), 250 pg (n = 2), 100 pg (n = 2), 50 pg (n = 2) and 10 pg (n = 2) were used as input for the WT-Ovation One-Direct system (NuGen Technologies, Inc). All cDNA reactions were purified via Zymo Research Clean and Concentrator™-25 or Qiagen RNeasy MinElute Cleanup kits (WT-Ovation FFPE V2 and WT-Ovation One-Direct systems respectively) as recommended. All purified cDNA probes were assessed for quantity and quality using the Agilent 2100 Bioanalyzer and the Nanodrop-8000 RNA Nano chips. FL-Ovation™ cDNA Biotin Module V2 (NuGEN) was used for fragmentation and biotin labelling of 5 µg of cDNA and used for subsequent hybridisation to Affymetrix HGU133 Plus 2.0 microarrays.

Both kits were assessed over a range of input levels within their own working ranges. Previously, studies using HUVEC RNA titrations had suggested that sscDNA probe yield and quality would be compromised below 200 pg in the WT-Ovation FFPE RNA amplification system (data not shown). HUVEC RNA dilutions were split (when input overlapping across systems), used as input template for both systems and processed in parallel (Figure 2).

**Figure 3:**
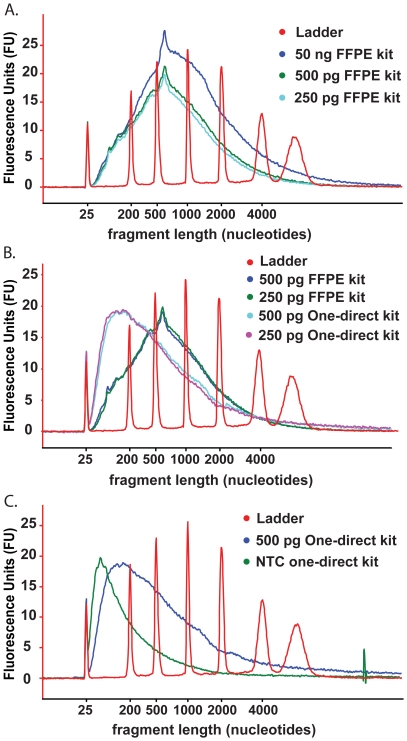
Bioanalyser electropherograms of cDNA probes generated using the WT-Ovation FFPE RNA amplification system V2 and the WT-Ovation One-Direct RNA amplification system. cDNA quality assessed by distribution of size with the x-axis representing polynucleotide length and the y-axis representing arbitrary signal intensity fluorescence units. Electropherograms of representative cDNA from each WT-Ovation system are shown. (**A**) WT-Ovation FFPE system sscDNA synthesised from 50 ng of RNA is distributed 500–1000 nucleotides. The sscDNA synthesised from 500 pg and 250 pg of RNA input in the WT-Ovation FFPE system show a similar distribution to the sscDNA synthesised from 50 ng of RNA. (**B**) The majority of dscDNA fragments synthesised in the WT-Ovation One-Direct system average in length at approximately 100–150 nucleotides. RNA input level does not influence polynucleotide length (500 and 250 pg input shown). A significant difference in polynucleotide distribution is observed in 500 pg and 250 pg input RNA reactions depending on which WT-Ovation system used for probe synthesis. (**C**) In the WT-Ovation One-Direct system, the dscDNA probes generated from a reaction containing RNA template is distinct from that generated in a parallel no template control.

The WT-Ovation FFPE RNA amplification system V2 is marketed for use with RNA derived from FFPE material, within a working range of 50 ng–100 ng input. For this reason 50 ng of high quality HUVEC RNA was used as a positive control for the performance of the system. Quality assessment shows that the sscDNA molecules generated from 50 ng of HUVEC RNA are of broad distribution in length, averaging approximately 500–1000 nucleotides, suggesting efficient sscDNA synthesis (Figure 3 A). It can be seen in Table 2 that the sscDNA yield from 50 ng of HUVEC RNA was in excess of the 5 µg required for microarray hybridisations. The performance of the WT-Ovation FFPE RNA amplification system at lower RNA input levels compares favourably in sscDNA yield (Table 2) to that obtained with 50 ng RNA input. All samples, even those at 250 pg input RNA, yield the required 5 µg of sscDNA probe, whilst maintaining efficient template amplification, as illustrated by the overlaying size distributions of the sscDNA from 500 pg or 250 pg input samples with that derived from 50 ng RNA (Figure 3 A).

**Table 2:** Quality metrics of cDNA probes generated from titrated HUVEC RNA using WT-Ovation FFPE and WT-Ovation One-Direct RNA amplification systems and hybridised to Affymetrix Human Genome U133 Plus 2.0 Arrays.

Amplificationkit	RNA input (pg)	cDNA yield (µg)	cDNA probe processed for GeneChip (µg)	% P calls	β-actin 3′/5′ ratio
FFPE	50000	9.93	5	61.0	1.95
FFPE	500 a	9.63	5	51.5	2.31
FFPE	500 b	9.39	5	50.9	2.28
FFPE	250 a	6.23	5	47.3	2.36
FFPE	250 b	8.32	5	47.3	2.57
One-Direct	500 a	16.27	5	50.0	5.87
One-Direct	500 b	16.33	5	48.3	4.27
One-Direct	250 a	17.19	5	46.6	4.61
One-Direct	250 b	14.47	5	44.4	4.58
One-Direct	100 a	14.78	5	38.9	4.30
One-Direct	100 b	13.69	5	39.2	4.01
One-Direct	50 a	13.99	5	34.0	4.91
One-Direct	50 b	13.99	5	36.0	3.01
One-Direct	10 a	13.62	5	19.2	7.21
One-Direct	10 b	13.18	5	18.3	5.30
One-Direct	NTC	9.36	5	2.3	5.87

RNA input, cDNA yield and mean quality metrics for microarrays following hybridisation of 5 µg of cDNA probes generated from either WT-Ovation FFPE or One-Direct RNA amplification systems. Quality metrics reflect Expression Console (Affymetrix) report data generated following MAS5.0 feature extraction. NTC  =  no template control. Technical duplicates were performed for each.

The WT-Ovation One-Direct kit is specifically intended for amplification of RNA within the 500–10 pg range for hybridisation to microarrays. Technical duplicates of HUVEC total RNA were processed, reproducibly yielding dscDNA probe quantities in excess of the 5 µg required for array hybridisation (Table 2). Compared to the WT-Ovation FFPE RNA amplification system the cDNA fragments generated are on average shorter with the WT-Ovation One-Direct system suggesting less efficiency in amplification from lower RNA input amounts (Figure 3 B). The no template control (NTC) reaction in the WT-Ovation amplification systems are known to generate non-specific product in the absence of template, which whilst this does not hybridise to Affymetrix GeneChips should be assessed in order that non-relevant cDNA does not comprise the major species in the hybridisation reactions (data not shown). The extent of the non-specific amplification can be seen in the NTC from the WT-Ovation One-Direct system, which generated in excess of 9 µg but which appears distinct from those synthesised from template-containing reactions with respect to size distribution and therefore is present at such levels only in reactions with no template (Figure 3 C). Importantly, sscDNA profiles from reactions of 10 pg RNA input and higher are similar, yet distinct from NTC reactions. All sscDNA probes were fragmented, biotin labelled and subjected to Affymetrix GeneChip hybridisation as outlined.

### Faithfulness of Microarray Measurements with minimal total RNA input

**Figure 4:**
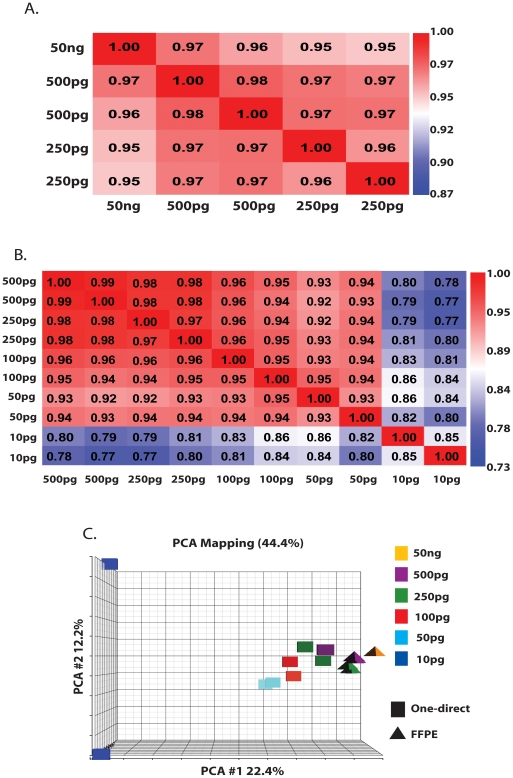
Pearson's Correlation of the signal (MAS5.0) obtained from Affymetrix GeneChips hybridised with cDNA probes synthesised using the NuGen WT-Ovation RNA amplification systems and the effect of reducing RNA input on the resultant Affymetrix GeneChips. (**A**) R^2^ values generated when comparing one GeneChip from 50 ng of input HUVEC RNA and duplicate GeneChips from 500 pg and 250 pg of input HUVEC RNA using MAS5.0 analysed data by Pearson's correlation of signal. (**B**) Duplicate GeneChips from 500 pg, 250 pg, 100 pg, 50 pg and 10 pg RNA input compared using MAS5.0 analysed data by Pearson's correlation of signal. **C**. Principal Component Analysis of all probe sets from GeneChips hybridised with cDNA probes from either WT-Ovation FFPE or WT-Ovation One-Direct RNA amplification systems.

Using the WT-Ovation FFPE system, the sscDNA probes generated from 50 ng of input RNA result in GeneChip metrics with 61% present calls (Table 2). Whilst these reduce as RNA input amount reduces, they remain at an appreciably high 51±0.48% for 500 pg and 47+0.04% for 250 pg input (mean ± standard deviation). β-actin and GAPDH 3′/5′ ratios (<2.57 and <1.07 respectively) indicate efficient amplification of the RNA template at all levels of input with no observed 3′ over-bias (Table 2). The impact of reducing RNA input level on the number of probe sets detected and how this is affected by transcript abundance can be observed by filtering probe sets by signal intensity (Table 3). It is clear that only a small reduction in total number of probe sets occurs with decreasing RNA input levels and the retention of probe sets is equally high regardless of overall abundance, as visualised by signal intensity (Table 3). The high reproducibility of all arrays from the WT-Ovation FFPE system, irrespective of input level, is clear from Pearson's signal correlation coefficients of no lower than 0.95 (MAS5.0) for all GeneChips, across a 200-fold range of input RNA (Figure 4 A).

**Table 3:** Impact of decreasing RNA input amounts on overall probe sets detected at varying transcript abundances by the WT-Ovation FFPE and WT-Ovation One-Direct RNA amplification systems.

Signal Intensity	Ovation-FFPE	50 ng	500 pg	250 pg			
Log_2_>5		30982	30471	30088			
Log_2_>6		24190	23416	22863			
Log_2_>7		18758	18266	18011			
Log_2_>8		13684	13303	13240			
Log_2_>9		9076	8667	8651			

	One-Direct		500 pg	250 pg	100 pg	50 pg	10 pg
Log_2_>5			31648	31593	31813	32612	42023
Log_2_>6			22854	22352	21360	20256	20269
Log_2_>7			17362	17003	16254	15401	10855
Log_2_>8			12664	12514	12055	11753	7809
Log_2_>9			8518	8433	8263	8248	5887

The number of probe sets detected at each signal intensity level (as a measure of transcript abundance) is shown. Numbers are the mean of duplicate arrays for input of 500 pg and below. Reduction of input RNA amounts has a minimal impact on the overall numbers of probe sets detected, but which is more evident with the One-Direct system, with a greater number of more abundant transcripts not being detected with decreasing RNA input.

The WT-Ovation One-Direct dscDNA probes from 500 pg and 250 pg of RNA input result in present calls similar to that achieved with the WT-Ovation FFPE system at the same overlapping input amount (Table 2) (500 pg, 49+1.2% Vs 51±0.48%; 250 pg, 45.5±1.1% Vs 47±0.04%). Excellent concordance across probe generation systems is further evidenced by Pearson's signal correlation coefficients of 0.92–0.93 (MAS5.0) at 500 pg of RNA input and 0.91–0.92 (MAS5.0) at 250 pg (data not shown). At an input level of lower than 250 pg the WT-Ovation One-Direct system resulted in approximately 39%, 35% and 18% at 100 pg, 50 pg and 10 pg respectively (Table 2). β-actin 3′/5′ ratio are notably higher in the WT-Ovation One-Direct system compared to those obtained by WT-Ovation FFPE, perhaps due to the shorter size distribution of cDNAs, suggesting increased 3′ over-bias and under-representation of 5′ targets (Table 2). As anticipated the non-specific product synthesised in the NTC reaction for the WT-Ovation One-Direct system failed to significantly hybridise to the GeneChip (Table 2, 2.3% present calls). Despite this reduction with reducing RNA input the high reproducibility of the GeneChips from 500 pg to 50 pg in the WT-Ovation One-Direct system can be seen in the Pearson's signal correlation coefficients of ≥0.92 (MAS5.0) for all GeneChips (Figure 4 B). This is supported by similarities in the number of probe sets detected as present at varying intensity levels in all Genechips from 500 pg to 50 pg input (Table 3). It is apparent with the One-Direct system however that a greater number of more abundant transcripts are not detected as RNA input is further progressively decreased (10 pg), as might be expected at this low (essentially single-cell) level of input. The “drift” in GeneChip performance and RNA targets being reliably targeted with lowering RNA template inputs can be seen by Principle Components Analysis (Figure 4 C). However, a significant shift in segregation is apparent when using 10 pg of input RNA in the WT-Ovation One-Direct system (Figure 4 C), further evidenced with the Pearson's signal correlation coefficient falling to 0.79 (MAS5.0) (Figure 4 B).

**Figure 5:**
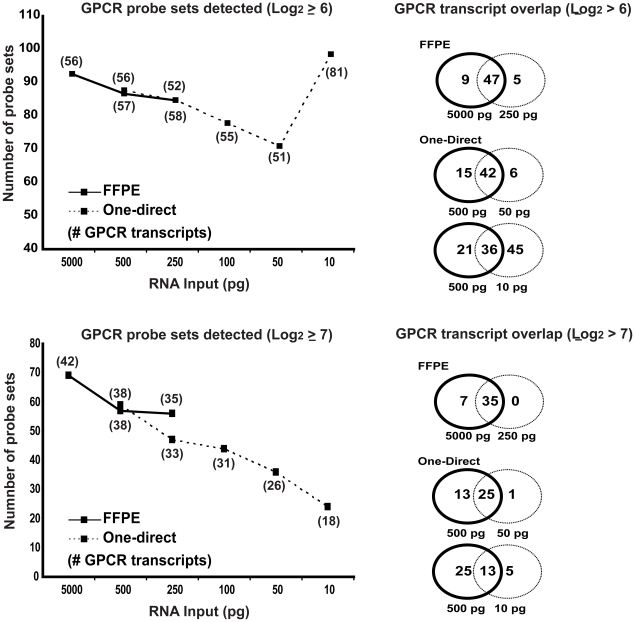
Low abundant probeset retention with decreasing RNA input levels. Data from the microarrays generated according to the workflow set out in Figure 2 was filtered for those probe sets representing a family of proteins, G protein-coupled receptors (GPCR), known to be of low abundance and difficult to detect using microarrays. The signal intensity was set at a threshold of Log_2_≥6 or Log_2_≥7 to ensure analysis of probe sets demonstrating strong hybridisation and robust signal. Data plotted is the number of GPCR probe sets present with differing RNA input levels and the number of genes that are represented in parenthesis below the data point. When duplicate GeneChips were available only the probe sets passing threshold in both duplicates were included. Venn diagrams illustrate the overlap of common genes between GeneChips from titrated RNA input levels for either WT-Ovation FFPE or WT-One-Direct RNA amplification systems.

To assess the retention of low abundant transcripts with decreasing RNA input levels and reduced probe set detection, microarray data was filtered for those probe sets representing a family of proteins, G protein-coupled receptors (GPCR), which are highly important for cellular signalling, however, known to be generally of low abundance and notoriously difficult to detect using microarray technology [37]. Using the WT-Ovation FFPE system the number of probe sets detected with 50 ng of input RNA is largely maintained when input is reduced to 250 pg (Figure 5). These probe sets represent 56 (log_2_≥6) and 42 (log_2_≥7) GPCR transcripts when using 50 ng and 52 (log_2_≥6) and 35 (log_2_≥7) transcripts from 250 pg input (Figure 5). Significantly, the overlap of common transcripts is impressively high, with 84% (log_2_≥6) and 83% (log_2_≥7) of transcripts detected at 50 ng of RNA still present when using 200 times less input of 250 pg RNA (Figure 5). When utilising the power of the WT-Ovation One-Direct system to further reduce the input RNA required for microarray analysis 51 (log_2_≥6) and 26 (log_2_≥7) GPCR transcripts are robustly detected at 50 pg of input RNA, representing 74% (log_2_≥6) and 66% (log_2_≥7) overlap with those detected when using 500 pg (Figure 5). Expression data generated from 10 pg of input RNA display a reduced number of transcripts detected (18 at log_2_≥7), with a 34% overlap with those detected at 500 pg input using the WT-Ovation One-Direct system (Figure 5). However, with a less conservative signal intensity filter of log_2_≥6 the number of GPCR probe sets detected increases from 87 probe sets with an input of 500 pg to 98 probe sets from 10 pg input. These 98 probe sets represent 81 genes, only 44% of which overlap with those found at 500 pg input. It is not clear what underpins this apparent anomaly of increased detection power with reduced input RNA. Whilst possibly representing false positive or inherent noise, it is also feasible that 10 pg input RNA (essentially representing single cell analysis) results in greater accessibility of the mRNA molecule for the subsequent enzymatic steps in the sscDNA generation procedure, at least for this class of transcripts.

### Effect of probe generation method on microarray performance of vascular endothelial biopsy samples

**Figure 6:**
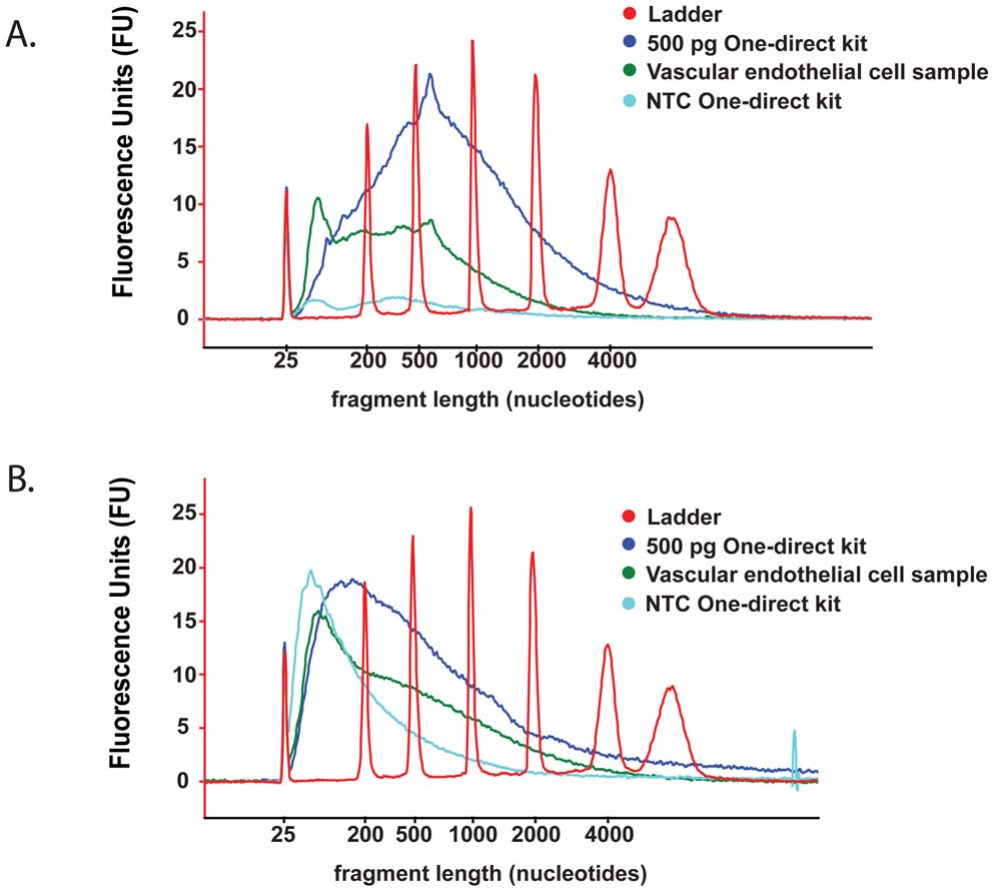
Bioanalyser electropherograms of cDNA probes synthesised by the WT-Ovation FFPE RNA amplification system V2 and the WT-Ovation One-Direct RNA amplification system using a vascular endothelial cell biopsy sample. cDNA quality assessed by distribution of size with the x-axis representing polynucleotide length and y-axis representing arbitrary signal intensity fluorescence units. Using a vascular endothelial cell biopsy sample a shift to shorter cDNA lengths is apparent compared to 500 pg total HUVEC RNA input in both the (**A**) WT-Ovation FFPE RNA amplification system V2 and the (**B**) WT-Ovation One-Direct RNA amplification system. NTC  =  no template control.

In order to determine the preferred approach for microarray analysis using vascular endothelial biopsies, RNA derived from a representative test sample was equally split and processed through both the WT-Ovation FFPE RNA amplification system V2 and the WT-Ovation One-Direct RNA amplification system. An input level of 300 pg of total RNA was chosen to allow comparison of both systems. Resultant yields of cDNA probe obtained from 300 pg of input RNA were 4.3 µg and 11.9 µg in the WT-Ovation FFPE and WT-Ovation One-Direct systems, respectively (Table 4). The sscDNA obtained using the WT-Ovation FFPE system fails to reach the recommended 5 µg of probe for fragmentation and hybridisation to Affymetrix GeneChips. The quality of the cDNA probes synthesised from both systems also displayed a reduction in size distribution when using clinical sample RNA compared to HUVEC RNA, associated with reduced efficiency of RNA amplification (Figure 6). To enable a direct comparison of hybridised material, 3.2 µg of cDNA probe was used from each system for fragmentation, labelling and hybridisation. In addition, a second hybridisation containing the recommended 5 µg of dscDNA from the WT-Ovation One-Direct kit was carried out as a further control. Despite the lower amount of cDNA probe hybridised, the WT-Ovation FFPE kit performed well by generating GeneChip quality metrics which include 30.5% present calls (Table 4). In contrast, the cDNA probe hybridised from the WT-Ovation One-Direct kit at both 3.2 µg and 5 µg failed to produce % present calls of above background level of hybridisation achieved by NTC (Table 4). Based on cDNA size distribution and subsequent microarray quality metrics the WT-Ovation FFPE system was chosen as the preferred technology for global transcriptomics of vascular endothelial biopsies collected and processed under the current conditions.

**Table 4:** Quality metrics from test vascular endothelial biopsy sample cDNA probes generated by the WT-Ovation FFPE and WT-Ovation One-Direct RNA amplification systems.

Amplification kit	cDNA probe yield (µg)	cDNA probe processed for GeneChip (µg)	% P calls	β-actin 3′/5′ ratio
FFPE	3.2	3.2	30.5	4.50
One-Direct	10.7	3.2	2.4	35.68
One-Direct	10.7	5	2.7	43.90

cDNA yield and microarray quality metrics generated from one vascular endothelial cell biopsy using either the WT-Ovation FFPE or One-Direct RNA amplification system. Quality metrics reflect Expression Console (Affymetrix) report data generated following MAS5.0 feature extraction.

### Efficacy of cDNA probe generation from vascular endothelial biopsy samples

**Figure 7:**
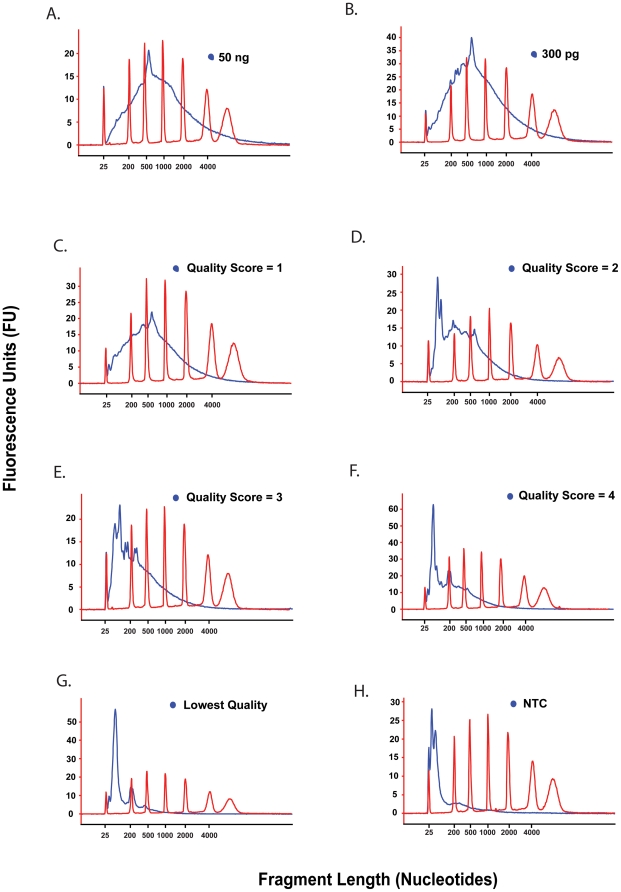
Assignment of “quality score” to sscDNA probes. Quality scores of 1–4 for sscDNA were assigned following visual assessment of distribution of polynucleotide size, with representative electropherograms shown here. (**A**) sscDNA synthesised from 50 ng total HUVEC RNA represents an amplification positive control of highest quality achievable. (**B**) sscDNA synthesised from 300 pg total HUVEC RNA amplification positive control of highest quality that could be expected from 300 pg of input RNA. (**C–G**) sscDNA synthesised from 300 pg of total RNA from a vascular endothelial biopsy sample. sscDNA has a electropherogram similar to that seen in the positive controls, peaking at over 500 nts and so was attributed a cDNA quality of 1 (C). sscDNAs progressively shorter in size distribution were designated quality scores of 2, 3 or 4 (D–G). sscDNAs designated lowest quality and therefore not hybridised to GeneChips (C) were of predominantly <200 nts in length. (**H**) No template control (NTC).

sscDNA probe generation using the WT-Ovation FFPE RNA amplification system V2 was carried out using RNA from vascular endothelial cell biopsy samples, with all inputs normalised to 300 pg RNA. With the assumption of an RNA content of 8 pg/cell, this equates to our global profiling being performed on the RNA equivalent of 37 purified endothelial cells. Whilst it is possible to generate array datasets from this range of input RNA material without the requirement to normalise RNA input for all samples, data outlined here clearly demonstrate that omission of normalisation of input quantities would likely introduce further magnitudes of variability (Figure 4 C). To maximise ability to capture of variability accounted for primarily by biological factors only, it was considered prudent to normalise input RNAs of all samples prior to array probe generation. The numbers of samples achieving the 300 pg RNA yield was 93 out of 114 (81.6%). sscDNA probes were generated in batches according to kit size (to avoid freeze-thaw cycles) and included a 50 ng and 300 pg HUVEC RNA positive control for each batch, together with a NTC. A summary of yields and quality of resultant sscDNA probes is outlined in Table 5. The yield from the NTC reactions consistently generated <3 µg of sscDNA, within the manufacturer's guidelines (Figure 7 H). The sscDNA yields from the 50 ng and 300 pg HUVEC RNA positive controls were in excess of the 5 µg typically required for GeneChip hybridisation. Quality assessment by Agilent Bioanalyzer shows that the sscDNA fragments generated from the 50 ng and 300 pg of HUVEC RNA positive controls were of broad distribution in length, averaging approximately 500–1000 nucleotides, suggesting efficient sscDNA synthesis (Figure 7 A, B). The average yield across all 93 clinical samples was 6.86±1.89 µg of sscDNA remaining following quality control assessment (Table 5). Of the 93 clinical samples utilised, 86% yielded over 5 µg considered the maximum probe required to proceed to hybridisation to GeneChips. Reducing the amount of sscDNA probe hybridised to the GeneChips to 4.5 µg was chosen to represent a balance between maximal cohort inclusion (91%) without significantly negatively impacting array quality.

**Table 5:** sscDNA probe yields for vascular endothelial biopsy samples, titrated HUVEC RNA positive controls and NTC's synthesised using the WT-Ovation FFPE RNA amplification system.

Sample Type	sscDNA yield(µg)	SD
50 ng HUVEC (n = 10)	18.52	1.76
300 pg HUVEC (n = 10)	9.12	2.49
NTC (n = 10)	1.32	0.20
Biopsy samples (n = 93)	6.86	1.89

Mean yields after quantification of sscDNA generated from vascular endothelial biopsy samples (n = 93). Positive control reactions using RNA input of 50 ng or 300 pg of HUVEC total RNA were run along side all batches of cDNA probe synthesis (n = 10). In each batch there was also a no template control reaction included. SD  =  Standard deviation.

A broad spectrum of sscDNA quality derived from vascular endothelial RNA was observed, with the size distribution of some cDNAs peaking at 500–1000 nucleotides as would be expected for highest quality probes, but others generated sscDNA peaking at less than 500 nts, representing less efficient synthesis. Twenty-one of the sscDNAs displayed a high level of short fragments (<200 nucleotides) deemed as non-specific amplification and failed to display a significant amount of efficient and template specific amplification so were not hybridised to GeneChips (Figure 7 G). By visual inspection of the 64 remaining sscDNA sample electropherograms an sscDNA quality score of 1–4 was attributed, based on average size distribution and extent of overlay with positive control electropherograms, with “1” representing highest quality (Figure 7 C–F). All cDNA probes were fragmented and biotin-labelled in preparation for hybridisation and all resulted in fragments of ∼50–200 nt (data not shown), as expected [36].

### Microarray performance of vascular endothelial biopsy samples

**Figure 8:**
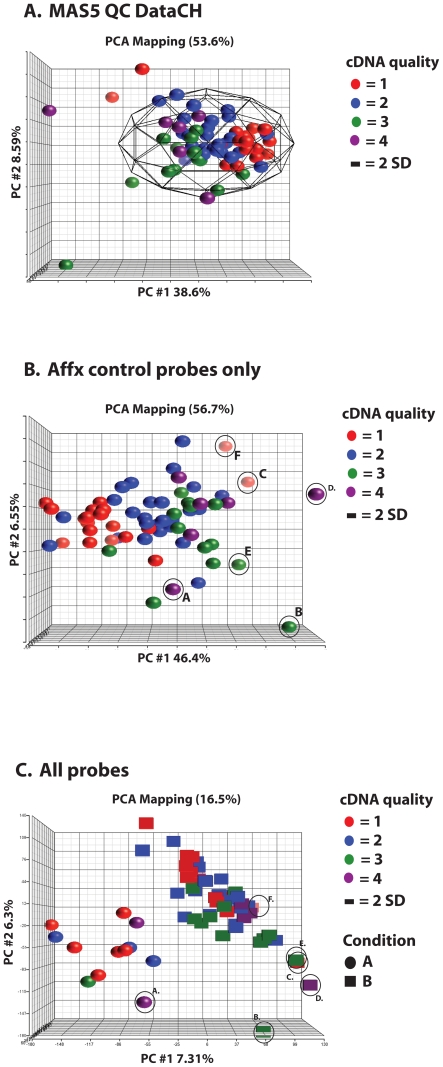
Assessing GeneChip quality control metrics and the effects of cDNA probe quality using Principle Components Analysis. (**A**) MAS5.0 QC data generated in Expression Console (Affymetrix) displayed using PCA illustrates the majority of GeneChips (n = 64) clustering together within 2 standard deviations of the group mean. The PCA is coloured according to the sscDNA quality score designated to each sample. Of the small number of samples lying out with the 2 SD boundary, sscDNA quality does not appear to be responsible for the variation shown, with this being contributed by other experimental factors. (**B**) PCA visualisation of Affx control probes only, coloured by sscDNA quality score shows that a significant proportion of the variation is driven by quality of the sscDNA probes. (**C**) PCA visualisation including all probes reveals the distinct sub-groups of the cohort according to expressed transcriptome patterns, regardless of cDNA quality.

Mean array quality control metrics were assessed for all 64 vascular endothelial biopsy samples hybridised to GeneChips. Mean % present calls of 32.0%±7.21% (mean ± SD) (Table 6) were achieved along with good control metrics such as background (29.6±1.28) and noise (0.86±0.09) suggesting efficient and consistent hybridisation of all arrays (Table 6). These were also associated with mean β-actin 3′/5′ ratios of 2.61±1.07 and GAPDH 3′/5′ ratios of 1.59±1.21 suggesting high quality and efficient amplification of RNA (Table 6). Principle Component Analysis of all quality control metrics following MAS5.0 feature extraction was carried out (Figure 8 A). In concordance with the MAQC established framework [38], designed to increase the consistency of microarray datasets, the majority of arrays in this study cluster within 2 standard deviations when assessed by quality metrics (Figure 8 A). Those arrays lying outside the 2 SD range were explained by either (a) high housekeeping gene 3–5′ ratios, suggesting reduce probe amplification efficiency or (b) lower signal intensity/lower spike-in control signal suggesting less efficient hybridisation. These “outlier” arrays samples remain out with the group clusters whether using all QC metrics, array AFFX control probes only or when using all probes (Figure 8 A, B, C). Principles component analysis focusing on the GeneChip control probes only demonstrates the lack of distinct populations of GeneChips caused by any experimental factors (Figure 8 B), providing evidence for the positive impact of the controlled reduction of variation introduced by technical parameters. Colouring of these data points by sscDNA quality however clearly illustrates the impact of this factor on variability within the dataset (Figure 8 B). Nonetheless, Figure 8C demonstrates clear segregation and capturing of biological variability between two of the sample groups studied here, the results of which will be presented in detail elsewhere.

**Table 6:** Quality metrics following hybridisation of sscDNA probes from vascular endothelial biopsy samples to Affymetrix Human Genome U133 Plus 2.0 arrays.

	Average Background	Average Noise	Scaling factor	% P calls	β-actin 3′/5′ ratio	GAPDH 3′/5′ ratio
Clinical samples (n = 64)	29.6±1.3	0.86±0.1	3.74±1.9	32.03±7.2	2.61±1.1	1.59±1.2

Quality metrics reflect Expression Console (Affymetrix) report data generated following MAS5.0 feature extraction. Values are mean ± standard deviation.

## Discussion

A great deal of progress has been made over the past few years in reducing the amount of input material required to carry out global transcriptional profiling using microarray technology. There are several reports of microarray datasets produced using laser capture micro-dissected material and from clinical biopsies which have yielded only nanograms of RNA material [19], [22]. However, there are few reports of successful global transcriptomics using input RNA within the picogram range [17], [24], [27], [39]. In addition, many of these reports are based on the titrated dilution of stock RNA solutions and not the “field” requirement of robust and successful arrays subsequent to recovery of picograms of RNA from minimal cell numbers [17], [24]. There are cases, such as in this study, where a clinical biopsy yields material estimated to be <1000 cells [29], making recovery of adequate amounts of RNA and subsequent transcriptional profiling significantly challenging.

RNA extraction methods shown to successfully yield good quality RNA of sufficient amounts from thousands or millions of cells are of course not necessarily optimised for use when using samples containing hundreds of cells. We observed several steps in traditional RNA extraction workflows to have a detrimental effect on RNA yield from minimal cell numbers, for example passing cell lysates through homogenisation matrices or carrying out on-column DNAse I treatments. The negative impact of these workflow components on RNA yield becomes evident only when using lower amounts of starting material. However, by altering these workflows and using titrated cell numbers we have succeeded in establishing an optimal RNA extraction process which yields approximately 2.5 times higher RNA yields when utilising below 3000 cells. Isolating RNA from cell populations such as these is challenging and whilst an average estimate of RNA content per cell may be in the range of 10 pg, depending on the cell type, is still not guaranteed to yield amounts of RNA within the nanogram range. To ensure maximum exploitation of the minimal cell samples available to us, we also sought to establish an RNA amplification method capable of producing robust array hybridisation probes, resulting in data capable of capturing biological information of interest, from RNA within the picogram range.

The WT-Ovation FFPE RNA amplification system is specifically designed for use with RNA of reduced integrity from FFPE material. This system employs random priming in addition to PolyA priming and incorporates a secondary SPIA linear amplification step. In this study we proposed to exploit this increased amplification power for use with minimal input RNA amounts extracted from any tissue matrix. The FFPE system demonstrated that 250 pg of input RNA yields the same high quality cDNA probes and GeneChips with high correlation (R_2_>0.95) to those obtained using 50 ng of input RNA. This translated to retention of 84% of the genes detected at 50 ng input remaining as a robust signal at 250 pg input when studying a family of genes (GPCRs) known to be of low abundance and difficult to detect by conventional microarray analysis. When processing clinical biopsies containing minimal cell numbers good quality cDNA and array metrics were obtained, although a reduction in sscDNA yield was observed. However, this reflected loss of synthesised material rather than poor overall synthesis, resulting from sscDNA purification schema employed at that time and which has since been changed to an alternative.

Further opportunity for increased amplification power when using RNA input within the picogram range is offered by the One-Direct RNA amplification system, designed for use with high quality RNA ranging from 500 pg down to 10 pg of input. We assessed this using titrated high quality RNA within the kits working range and found that reducing RNA input in this kit does not influence the quality of cDNA probe generated, with all samples from 500 pg to 10 pg producing similar populations of cDNA probe size distribution. The cDNA probes generated at all input levels using the One-Direct system did consist of shorter probe fragments than those generated from at little as 250 pg using the FFPE system. However, this does not appear to significantly affect the performance of the subsequent microarrays as illustrated by the similarities in the microarray metrics from the FFPE and One-Direct system at the same levels of input RNA. Within the One-Direct system high correlation between GeneChips from decreasing input material can been seen from 500 pg down to 50 pg of input (R_2_>0.92). Unfortunately, as might be expected we did not observe this high level of performance when using 10 pg of input RNA as these samples generate R_2_ values of 0.77–0.86 when compared to higher input levels, with apparent loss of detection of higher abundance transcripts. We conclude therefore that the One-Direct system can further push the lower limits of material required for probe synthesis for microarray experiments to 50 pg of input when using high quality RNA, without significant impact on resulting information generated. Nonetheless, 10 pg of input RNA still results in generation of arrays from which a large amount of transcriptional information can be derived (approximately 19% present calls). However, it should be noted that variation in input RNA results in differences in the transcriptional information captured by array, and which is particularly evident at low input levels. Therefore, whilst quantification of such low levels following primary RNA isolation is challenging, we recommend that this is performed to allow subsequent input normalisation (as outlined in the current study) and eventual maximisation of ability to interrogate variability due to biology rather than technical parameters.

Whilst not exploited here, a further distinct advantage of the One-Direct system is the option of preparing microarray probes directly from cell lysis of minimal cell numbers without prior RNA purification. The capability of direct cell lysis has the potential to negate the need for RNA extraction and avoid the inevitable loss of RNA accompanying these workflows. However, RNA extraction methods specifically optimised for minimal cell numbers remain relevant as not all collection techniques provide the material in the appropriate conditions/volume required for this direct cell lysis step. For example, the vascular endothelial cell biopsies used in this study require an immuno-bead positive enrichment stage within the cell collection workflow involving a higher liquid volume than that required to commence the One-Direct system at the cell lysis stage. Future efforts will focus on alternative endothelial cell collection schema to exploit the low working range of the One-Direct kit. Following on from our work with the One-Direct system using high quality RNA at 50 pg or above and the additional opportunity to process some types of minimal cell samples with direct cell lysis we believe that this technology not only further lowers the limits of input material needed to carry out microarray experiments but also increases the sample types which can be robustly interrogated by global expression profiling. Nonetheless, based on our observations here, it is still desirable if possible to begin with a known, normalised, amount of input RNA, such that inherent variation is limited to that of biological origin.

Similarly, we believe that assessment of the performance of array probe generation workflows using control RNA titrations rather than bona fide material of interest do not always realistically mirror the experimental conditions which will be met.

Due to the successful performance of the FFPE system and the majority of clinical samples yielding over the lower limit of 250 pg input RNA with this system we processed our vascular endothelial cell biopsies on to microarrays. The average percentage of present calls on these GeneChips was 32%, along with acceptable quality for all other control metrics. Whilst still representing a relatively high number of transcripts being interrogated, this percentage of present probes is lower than we would expect when using titrated good quality RNA to the same low levels. Previous reports have suggested that samples of <1000 cells can only generate reliable expression data for the most abundantly expressed genes, thereby leading to a biased dataset [40]. Importantly, we have demonstrated here that the number of probe sets detected shows a high level of retention with lowering RNA input levels in two different probe generation methods, impacting only the most abundant transcripts at low input levels in the One-Direct system.

In the present study we have established an RNA extraction method specifically designed to isolate the maximum RNA from minimal cell numbers, and have utilised recent developments in isothermal linear amplification methods to generate reliable and robust microarray data from these minimal cell clinical samples, which we estimate to have been perfomed on the RNA equivalent of approximately 37 cells. Several reports have supported the use of an isothermal amplification technique for cDNA probe generation for use with microarrays [17], [24], [27], [35], [36]. These have included reports for diluted RNA down to 250 pg of RNA input [24], [39]. This study has further advanced these findings by demonstrating the use of WT-Ovation technology at low levels of input to generate a dataset of global transcriptional profiling from clinical samples following RNA extraction from minimal cell numbers. Our work with the One-Direct RNA amplification system suggests that this lower limit of input can now be further reduced to 50 pg of good quality RNA without obvious impact on data quality or loss of lower abundant genes and can be utilised at 10 pg input RNA, albeit with expected and accepted loss of transcriptome information. The One-Direct system may be exploitable for some clinical sample types by using the direct cell lysis workflow offered to eradicate the need for RNA extraction workflows. We also demonstrate the successful global transcriptional profiling of biopsies containing minimal cell numbers which have previously been limited to focussed transcriptional interrogation. Whilst we have not embarked here on formal validation of identified transcriptional differences to demonstrate the validity of our methodologies as has been done by others [39], our analysis strategy of utilising the entire transcriptome to inform correlation co-efficients rather than filtered gene sets has been previously demonstrated to be optimal [39]. Furthermore, the transcriptional changes we have observed here are greater than 5000 which pass multiple test correction and two-fold change filtering, as evidenced by the striking separation of phenotypic groups in PCA. In our experience of post-array confirmation of similar sets of targets derived from sscDNA-based microarray studies, we have been able to confirm by qPCR the vast majority of these. Additionally, our analyses here suggest the ability of these procedures to allow interrogation of classes of rare transcripts, even at impressively low input RNA amounts, for which microarray technology is widely believed to be insufficiently sensitive. The ability to interrogate the global transcriptome of clinical samples containing limited cellular material has the potential to allow molecular profiling endeavours from previously inaccessible sources.

## Methods

### Cell culture

Low passage Human umbilical venous endothelial cell line (HUVEC) was obtained from Millipore and were maintained in EGM-2 BulletKit medium (Lonza, USA) according to the manufacturers recommendations at 37°C in 5% CO_2_ and 100% humidity. Cells were harvested and counted following growth to ∼80% confluency. Briefly, the media was removed from the flask and the cell monolayer was washed by pipetting 1 x HEPES Buffered Saline Solution (Lonza, USA) over the cells. Following removal of the 1 x HEPES Buffered Saline Solution, 1 ml of 1×0.05% Trysin/EDTA solution (Sigma-Aldrich) was added to the flask. Cells were loosened by placement in the incubator for 1 minute followed by the addition of 1 x Trypsin Neutralizing Solution and then suspension in 1 x HEPES Buffered Saline Solution. The cell suspension was centrifuged for 5 minutes at 220 x *g* and the cell pellet resuspended in 1 x HEPES Buffered Saline Solution. Following a second centrifugation the cell pellet was resuspended in fresh 1 x HEPES Buffered Saline Solution.

### Cell counting

Four 200 µl aliquots of the cell suspension were removed to 1.5 ml tubes. To each aliquot 200 µl of Solution A-100 (Chemometec A/A, Denmark) was added and each tube vortexed for 30 seconds. 200 µl of Solution B-100 was then added to each tube followed by vortexing for 30 seconds. The cell solution was analysed by a NucleoCounter (ChemoMetec A/S) following loading of each sample in to a Cell Counting Cartridge. All samples were counted for cell number in quadruplicate and diluted in 1 x HEPES buffer to the desired cell number (ranging from 3000 to 200 cells) in a final volume of 50 µl. To each 50 µl sample of known cell number, 300 µl of buffer RLT/2-mercaptoethanol (Qiagen) was added and all samples stored at −80°C prior to processing.

### Vascular endothelial biopsies

Venous endothelial cells were collected from healthy controls, patients with insulin resistance and coronary artery disease. The study was approved by the Tayside committee on Medical Ethics. All patients gave written informed consent and all experiments were carried out in accordance to the Helsinki Declaration.

Endothelial cells were collected after the volunteer had been laid supine for 30 minutes using a modified technique of venous endothelial biopsy as described by [29]. A 0.46 mm “J” tip Spring Wire Guide with Arrow Advancer (ARROW, Arrow International Inc., Germany) was used for EC sampling. Each wire was advanced through an 18 G cannula positioned in the forearm vein. The tip was advanced 10 cm and moved to and fro several times within the vein. The distal portion of the wire was then transferred to a 15 ml conical tube containing dissociation buffer (0.5% bovine serum albumin, 2 mM EDTA and 100 mcg/ml heparin in PBS, pH 7.4), kept at 4°C. Cells were removed from the distal portion of the wire by insertion into the “flush system”, attachment of syringes containing dissociation buffers (ECDS). This process was repeated for each wire using the same buffer contained within the syringe.

### Endothelial Cell separation

Each sample was split between 4 eppendorf tubes (approx 500 µl/tube) and 5 µl of dynabeads (Dynal Biotech Inc), pre-coated with mouse anti-human CD146 monoclonal antibody (Clone MAB16985, Millipore), was added to each tube. The tubes were incubated at 4°C for 25 mins with rotation before being placed in a magnetic rack. A magnet was used to collect the beads and the supernatant removed and discarded. The remaining beads were then washed twice with sterile ECDS (500 µl per wash), once with 500 µl sterile Hanks Balanced Salt Solution (HBSS) and twice with sterile Phosphate Buffered Saline (PBS) (500 µl per wash). After the first PBS wash the supernatant was discarded but after the second, approximately 50 µl of liquid was retained to resuspend the beads. Buffer RLT/2-mercaptoethanol (Qiagen, UK) was added to the bead suspension. Brief vortexing to disrupt the cells from the beads and then separation by 2 minutes on the magnet was carried out. Finally, the resultant lysate was transferred to a fresh, sterile eppendorf tube and stored at −80°C. The entire cell separation procedure was completed within 1 hour of venous cell biopsy.

### Total RNA isolation

Total RNA was isolated from 1×10^6^ HUVEC using the RNeasy Mini RNA isolation kit (Qiagen) according to manufacturer's instructions. Briefly, 350 µl HUVEC lysate samples were thawed following storage at −80°C. To ensure complete cell lysis the samples were vortexed vigorously for 10 seconds and pulse spun. An equal volume (350 µl) of 70% ethanol was added to the cell lysate and mixed by pipetting. The entire sample was applied to an RNeasy Mini Spin Column in a 2 ml collection tube. The RNeasy Mini Spin Columns were centrifuged for 15 seconds at 16,300 x g. The flow-through was re-applied to the same RNeasy Mini Spin Column and the centrifugation repeated. 350 µl of buffer RW1 was applied to each RNeasy Mini Spin Columns followed by centrifugation for 15 seconds at 16,300 x g. A DNase I incubation mix was prepared by combining 10 µl of DNase I and 70 µl of Buffer RDD per sample. DNase treatment was performed by pipetting 80 µl of DNase I incubation mix directly on to the RNeasy Mini Spin column membrane and incubating at room temperature for 15 minutes. To wash the RNeasy Mini Spin Column, 350 µl of Buffer RW1 was added and centrifuged for 15 seconds at 16,300 x *g* followed by 500 µl of Buffer RPE and centrifugation for 2 minute at 16,300 x *g*. An additional centrifugation for 1 minute at 16,300 x *g* was carried out to dry the column membrane. RNA was eluted into fresh 1.5 ml microcentrifuge tubes using 50 µl of RNase-free water followed by centrifugation for 1 minute at 16,300 x *g*.

When isolating RNA from titrated minimal cell numbers (3000 – 200) total RNA was isolated using the RNeasy Micro RNA isolation kit (Qiagen) following the manufacturers instructions (process A) or with modifications (process B) (Figure 1A). RNA isolation from vascular endothelial biopsies was also carried out using the RNeasy Micro RNA isolation kit (Qiagen) following process B (Figure 1A).

When following the manufacturer's instructions (process A) the cells were suspended and lysed by vortexing vigorously for 10 seconds and pulse spun. Sample were homogenised by pipetting the cell lysate directly onto a QIAshredder Spin Column (QIagen) placed in a 2 ml collection tube and centrifuged for 2 minutes at 16,300 x *g*. An equal volume (350 µl) of 70% ethanol was added to the cell lysate and mixed by pipetting. The entire sample was applied to an RNeasy MinElute Spin Column in a 2 ml collection tube. The RNeasy MinElute Spin Columns were centrifuged for 15 seconds at 16,300 x *g*. The flow-through was re-applied to the same RNeasy MinElute Spin Column and the centrifugation repeated. 350 µl of buffer RW1 was applied to each RNeasy MinElute Spin Column followed by centrifugation for 15 seconds at 16,300 x g. A DNase I incubation mix was prepared by combining 10 µl of DNase I and 70 µl of Buffer RDD per sample. DNase treatment was performed by pipetting 80 µl of DNase I incubation mix directly on to the RNeasy Mini Spin column membrane and incubating at room temperature for 15 minutes. To wash the RNeasy Mini Spin Column, 350 µl of Buffer RW1 was added and centrifuged for 15 seconds at 16,300 x *g* followed by 500 µl of Buffer RPE and centrifugation for 15 seconds at 16,300 x *g*. A further 500 µl of 80% Ethanol was added and the column centrifuged for 2 minutes at 16,300 x *g*. An additional centrifugation for 5 minutes at 16,300 x *g* was carried out to dry the column membrane. RNA was eluted into fresh 1.5 ml microcentrifuge tubes by adding 20 µl of RNase-free water on to the membrane followed by centrifugation for 1 minute at 16,300x *g*. The eluate was reapplied to the same RNeasy MinElute Spin Column and the centrifugation repeated. All samples were stored at −80°C prior to further processing.

Total RNA from minimal cell numbers was also extracted using the RNeasy Micro RNA isolation kit with the following modifications (process B) (Figure 1A). Cells were suspended and lysed by vortexing vigorously for 10 seconds and pulse spun. An equal volume (350 µl) of 70% ethanol was added to the cell lysate and mixed by pipetting. The entire sample was applied to an RNeasy MinElute Spin Column in a 2 ml collection tube. The RNeasy MinElute Spin Columns were centrifuged for 15 seconds at 16,300 x *g*. The flow-through was re-applied to the same RNeasy MinElute Spin Column and the centrifugation repeated. 700 µl of buffer RW1 was applied to each RNeasy MinElute Spin Column followed by centrifugation for 15 seconds at 16,300 x g. To wash the RNeasy MinElute Spin Column, 500 µl of Buffer RPE was added and centrifuged for 15 seconds at 16,300 x *g* followed by 500 µl of 80% Ethanol and centrifugation for 2 minute at 16,300 x *g*. An additional centrifugation for 5 minutes at 16,300 x *g* was carried out to dry the column membrane. RNA was eluted into fresh 1.5 ml microcentrifuge tubes by incubating 20 µl of RNase-free water on the membrane for 1 minute followed by centrifugation for 1 minute at 16,300 x *g*. The eluate was reapplied to the same RNeasy MinElute Spin Column and the incubation and centrifugation repeated.

Genomic DNA (gDNA) elimination was carried out on the total RNA samples from minimal cell numbers by gDNA Wipeout buffer treatment (Quantitect Reverse transcription kit, Qiagen). Firstly, the 20 µl RNA samples were dried down in a Concentrator 5301 (Eppendorf) without heat for 9 minutes to obtain a final volume of 12 µl. To each concentrated RNA sample 2 µl of gDNA Wipeout buffer was added and incubated at 40°C for 5 minutes.

Total RNA was recovered from the gDNA elimination reaction using the RNeasy Micro kit (Qiagen) RNA clean up protocol. Briefly, 86 µl of RNase-free water was added to the gDNA elimination reactions. 350 µl of buffer RLT/2-mercaptoethanol was added and mixed by pipetting. 250 µl of 100% Ethanol was added and mix by pipetting. The entire sample was applied to an RNeasy MinElute Spin Column in a 2 ml collection tube. The RNeasy MinElute Spin Columns were centrifuged for 15 seconds at 16,300 x *g*. The flow-through was re-applied to the same RNeasy MinElute Spin Column and the centrifugation repeated. 500 µl of buffer RPE was applied to each RNeasy MinElute Spin Column followed by centrifugation for 15 seconds at 16,300 x *g*. To wash the RNeasy MinElute Spin Column, 500 µl of 80% Ethanol and added and the columns centrifuged for 2 minute at 16,300 x *g*. An additional centrifugation for 5 minutes at 16,300x *g* was carried out to dry the column membrane. RNA was eluted into fresh 1.5 ml microcentrifuge tubes by incubating 20 µl of RNase-free water on the membrane for 1 minute followed by centrifugation for 1 minute at 16,300 x *g*. The eluate was reapplied to the same RNeasy MinElute Spin Column and the incubation and centrifugation repeated. All samples were stored at −80°C prior to further processing.

### Total RNA quantification

The quantity of total RNA from 1×10^6^ HUVEC was measured using a Nanodrop ND-8000 spectrophotometer (Nanodrop Technologies, Wilmington, DE). RNA quality was assessed in part by OD260/280 ratios but primarily by use of an Agilent 2100 Bioanalyzer (Agilent Technologies, Palo Alto, CA).

The quantity of total RNA from titrated minimal cell numbers and vascular endothelial biopsies was measured following reverse transcription of the RNA to cDNA and subsequent real-time quantitative PCR along with a calibration curve of RNA from the same cells. RNA reverse transcription was carried out using the Quantitect Reverse Transcription Kit (Qiagen) following the manufacturers instructions. Briefly, both unknown samples and standard curve samples made from HUVEC RNA dilutions of known concentration ranging from 500 ng–1 pg RNA were made up to 12 µl volume using RNase-free water. When measuring RNA quantity from HUVEC titrated minimal cell numbers, all of the isolated RNA was used in the Quantitect Reverse Transcription Kit (Qiagen). Following RNA isolation optimisation using either HUVEC titrated minimal cell numbers or vascular endothelial biopsies, 10% of isolated RNA was used for reverse transcription. gDNA Wipeout buffer (7 x) was added to each sample and mixed by pipetting. All samples were incubated at 42°C for 5 minutes and placed immediately on ice. A reverse transcription reaction was carried out by adding 1 µl of RT enzyme, 4 µl of Quantiscript buffer (5 x) and 1 µl of RT primer mix to each sample followed by incubation at 42°C for 20 minutes. The reaction was halted by a further incubation at 95°C for 3 minutes. All reaction were stored at −20°C prior to further processing.

Quantitect cDNA samples generated from minimal cell numbers and vascular endothelial biopsies or standard curve RNA dilutions were diluted 1∶25 or 1∶100, respectively for input in the real-time quantitative PCR. All quantification was carried out using β-actin as the target gene for amplification. SybrGreen (Bio-rad) along with β-actin primers used for quantification of HUVEC titrated minimal cells and vascular endothelial cell biopsy samples. Forward primer: 5-GGACTTCGAGCAAGAGATGG-3 and Reverse primer: 5-AGCACTGTGTTGGCGTACAG-3. PCR master mix was loaded to each well and the plate centrifuged at 2,000 x *g* for 20 seconds and run on a 7900HT (ABI).

### Microarray probe generation

Preparation of cDNA SPIA probes for microarray hybridisation, was carried out with varying amounts of total HUVEC RNA (50 ng–10 pg) or vascular endothelial cell samples using NuGEN Technologies (California, USA) WT-Ovation™ FFPE RNA amplification system V2 or WT-Ovation™ One-Direct RNA amplification system according to the manufacturers' instructions. cDNA probes generated using the WT-Ovation™ FFPE RNA amplification system V2 from RNA isolated from titrated minimal cell samples were purified prior to quantification using either the DNA clean and concentrator system (Zymo) or QIAquick PCR purification kit (Qiagen). All cDNA generated using the WT-Ovation™ One-Direct RNA amplification system was purified using the MinElute system (Qiagen) as directed by Nugen Technolgies, Inc. All cDNA probes generated from vascular endothelial biopsy samples were purified using the QIAquick PCR purification kit (Qiagen). The vascular endothelial cell biopsy samples were all processed for cDNA probe generation in batches including positive controls of 50 ng and 300 pg HUVEC total RNA and a no template control reaction. Synthesised cDNA probe concentration was measured by Nanodrop ND-8000 (Nanodrop Technologies, Wilmington, DE, USA). The NuGEN Technologies FL-Ovation™ cDNA Biotin Module V2 was used for cDNA fragmentation and biotin labelling of amplified cDNA for subsequent hybridisation to the microarrays (Affymetrix, Santa Clara, CA, USA). The quality of the cDNA probes was assessed before and after fragmentation with the Agilent 2100 Bioanalyzer using RNA Nano chips (Agilent Technologies, Santa Clara, CA, USA).

### Affymetrix GeneChips

cDNA probes generated from NuGen Technologies RNA amplification systems were hybridised to Affymetrix Human Genome U133 Plus 2.0 Arrays (Affymetrix, Santa Clara, CA, USA) as described in the Affymetrix Expression Analysis Technical Manual. Briefly, 5 µg (HUVEC) or 4.5 µg (vascular endothelial biopsy samples) of fragmented and labelled cDNA, together with spiked hybridisation controls (GeneChip Expression 3′ Amplification Reagents – hybridisation controls), was hybridised for 18 hrs at 45°C in a rotating oven. Following hybridisation GeneChip washing and staining was performed using the GeneChip Hybridisation Wash and Stain kit (Affymetrix, Santa Clara, CA, USA) on an Affymetrix GeneChip Fluidics Station 450 using the appropriate fluidic script for the U133 Plus 2.0 microarrays with cDNA (FS450-0004). GeneChips were scanned immediately following staining in an Affymetrix GeneChip Scanner 3000 (Affymetrix, Santa Clara, USA). MIAME-compliant array data can be accessed via Gene Expression Omnibus (GEO), GSE21723.

### Data analysis

Report files summarising the quality of target and control detection for each microarray were generated by GeneChip Operating Software Version 1.4 (GCOS) using the MAS5.0 algorithm (Affymetrix, Santa Clara, CA, USA). Quality control analysis was performed using Expression Console (Affymetrix) and Partek Genomics Suite. Correlation between GeneChips was assessed by Pearson's Correlation on the signal from all probes following MAS5.0 data extraction. Principal component analyses (PCA) were carried out using Partek Genomics Suite on MAS5.0 normalised data followed by Log_2_ transformation.

## References

[1] Bos PD, Zhang XH, Nadal C, Shu W, Gomis RR (2009). Genes that mediate breast cancer metastasis to the brain.. Nature.

[2] Chee M, Yang R, Hubbell E, Berno A, Huang XC (1996). Accessing genetic information with high-density DNA arrays.. Science.

[3] Pomeroy SL, Tamayo P, Gaasenbeek M, Sturla LM, Angelo M (2002). Prediction of central nervous system embryonal tumour outcome based on gene expression.. Nature.

[4] Cobleigh MA, Tabesh B, Bitterman P, Baker J, Cronin M (2005). Tumor gene expression and prognosis in breast cancer patients with 10 or more positive lymph nodes.. Clin Cancer Res.

[5] Glas AM, Floore A, Delahaye LJ, Witteveen AT, Pover RC (2006). Converting a breast cancer microarray signature into a high-throughput diagnostic test.. BMC Genomics.

[6] Eberwine J, Yeh H, Miyashiro K, Cao Y, Nair S (1992). Analysis of gene expression in single live neurons.. Proc Natl Acad Sci U S A.

[7] Duggan DJ, Bittner M, Chen Y, Meltzer P, Trent JM (1999). Expression profiling using cDNA microarrays.. Nat Genet.

[8] Schena M, Shalon D, Davis RW, Brown PO (1995). Quantitative monitoring of gene expression patterns with a complementary DNA microarray.. Science.

[9] Assersohn L, Gangi L, Zhao Y, Dowsett M, Simon R (2002). The feasibility of using fine needle aspiration from primary breast cancers for cDNA microarray analyses.. Clin Cancer Res.

[10] Sotiriou C, Powles TJ, Dowsett M, Jazaeri AA, Feldman AL (2002). Gene expression profiles derived from fine needle aspiration correlate with response to systemic chemotherapy in breast cancer.. Breast Cancer Res.

[11] Symmans WF, Ayers M, Clark EA, Stec J, Hess KR (2003). Total RNA yield and microarray gene expression profiles from fine-needle aspiration biopsy and core-needle biopsy samples of breast carcinoma.. Cancer.

[12] Marcus JS, Anderson WF, Quake SR (2006). Microfluidic single-cell mRNA isolation and analysis.. Anal Chem.

[13] Toriello NM, Douglas ES, Thaitrong N, Hsiao SC, Francis MB (2008). Integrated microfluidic bioprocessor for single-cell gene expression analysis.. Proc Natl Acad Sci U S A.

[14] Tang F, Barbacioru C, Wang Y, Nordman E, Lee C (2009). mRNA-Seq whole-transcriptome analysis of a single cell.. Nat Methods.

[15] Bak M, Conley L, Hedegaard J, Larsen LA, Sorensen P (2006). Evaluation of two methods for generating cRNA for microarray experiments from nanogram amounts of total RNA.. Anal Biochem.

[16] Klur S, Toy K, Williams MP, Certa U (2004). Evaluation of procedures for amplification of small-size samples for hybridization on microarrays.. Genomics.

[17] Singh R, Maganti RJ, Jabba SV, Wang M, Deng G (2005). Microarray-based comparison of three amplification methods for nanogram amounts of total RNA.. Am J Physiol Cell Physiol.

[18] Viale A, Li J, Tiesman J, Hester S, Massimi A (2007). Big results from small samples: evaluation of amplification protocols for gene expression profiling.. J Biomol Tech.

[19] King C, Guo N, Frampton GM, Gerry NP, Lenburg ME (2005). Reliability and reproducibility of gene expression measurements using amplified RNA from laser-microdissected primary breast tissue with oligonucleotide arrays.. J Mol Diagn.

[20] Oudes AJ, Campbell DS, Sorensen CM, Walashek LS, True LD (2006). Transcriptomes of human prostate cells.. BMC Genomics.

[21] Guglielmelli P, Zini R, Bogani C, Salati S, Pancrazzi A (2007). Molecular profiling of CD34+ cells in idiopathic myelofibrosis identifies a set of disease-associated genes and reveals the clinical significance of Wilms' tumor gene 1 (WT1).. Stem Cells.

[22] Peterkova M, Koutna I, Tesarova L, Potesilova M, Kozubek M (2009). Microarray analysis using a limited amount of cells.. Folia Biol (Praha).

[23] Bai X, Huang M, Wu J, Huang X, Yan L (2008). Development and characterization of a novel method to analyze global gene expression profiles in endothelial cells derived from primary tissues.. Am J Hematol.

[24] Clement-Ziza M, Gentien D, Lyonnet S, Thiery JP, Besmond C (2009). Evaluation of methods for amplification of picogram amounts of total RNA for whole genome expression profiling.. BMC Genomics.

[25] Kim CY, Kuehn MH, Clark AF, Kwon YH (2006). Gene expression profile of the adult human retinal ganglion cell layer.. Mol Vis.

[26] Hunter SM, Mansergh FC, Evans MJ (2008). Optimization of minuscule samples for use with cDNA microarrays.. J Biochem Biophys Methods.

[27] Shearstone JR, Allaire NE, Campos-Rivera J, Rao S, Perrin S (2006). Accurate and precise transcriptional profiles from 50 pg of total RNA or 100 flow-sorted primary lymphocytes.. Genomics.

[28] Dobson AT, Raja R, Abeyta MJ, Taylor T, Shen S (2004). The unique transcriptome through day 3 of human preimplantation development.. Hum Mol Genet.

[29] Colombo PC, Ashton AW, Celaj S, Talreja A, Banchs JE (2002). Biopsy coupled to quantitative immunofluorescence: a new method to study the human vascular endothelium.. J Appl Physiol.

[30] Clarkson P, Mullen MJ, Donald AE, Powe AJ, Thomson H (2001). The effect of amlodipine on endothelial function in young adults with a strong family history of premature coronary artery disease: a randomised double blind study.. Atherosclerosis.

[31] Han SH, Gerber TC, Suwaidi JA, Eeckhout E, Lennon R (2009). Relationship between coronary endothelial function and coronary calcification in early atherosclerosis.. Atherosclerosis.

[32] Lerman A, Zeiher AM (2005). Endothelial function: cardiac events.. Circulation.

[33] Colombo PC, Banchs JE, Celaj S, Talreja A, Lachmann J (2005). Endothelial cell activation in patients with decompensated heart failure.. Circulation.

[34] Onat D, Jelic S, Schmidt AM, Pile-Spellman J, Homma S (2007). Vascular endothelial sampling and analysis of gene transcripts: a new quantitative approach to monitor vascular inflammation.. J Appl Physiol.

[35] Kurn N, Chen P, Heath JD, Kopf-Sill A, Stephens KM (2005). Novel isothermal, linear nucleic acid amplification systems for highly multiplexed applications.. Clin Chem.

[36] Barker CS, Griffin C, Dolganov GM, Hanspers K, Yang JY (2005). Increased DNA microarray hybridization specificity using sscDNA targets.. BMC Genomics.

[37] Hansen A, Chen Y, Inman JM, Phan QN, Qi ZQ (2007). Sensitive and specific method for detecting G protein-coupled receptor mRNAs.. Nat Methods.

[38] Shi L, Reid LH, Jones WD, Shippy R, Warrington JA (2006). The MicroArray Quality Control (MAQC) project shows inter- and intraplatform reproducibility of gene expression measurements.. Nat Biotechnol.

[39] Lang JE, Magbanua MJ, Scott JH, Makrigiorgos GM, Wang G (2009). A comparison of RNA amplification techniques at sub-nanogram input concentration.. BMC Genomics.

[40] Nygaard V, Holden M, Loland A, Langaas M, Myklebost O (2005). Limitations of mRNA amplification from small-size cell samples.. BMC Genomics.

